# Reducing the Impact of Sensor Orientation Variability in Human Activity Recognition Using a Consistent Reference System

**DOI:** 10.3390/s23135845

**Published:** 2023-06-23

**Authors:** Manuel Gil-Martín, Javier López-Iniesta, Fernando Fernández-Martínez, Rubén San-Segundo

**Affiliations:** Speech Technology and Machine Learning, Information Processing and Telecommunications Center, E.T.S.I. de Telecomunicación, Universidad Politécnica de Madrid, 28040 Madrid, Spain; javier.lopeziniesta.diazdelcampo@alumnos.upm.es (J.L.-I.); fernando.fernandezm@upm.es (F.F.-M.);

**Keywords:** human activity recognition, gravity estimation, sensor-orientation-independent, forward movement direction, wearable sensors, acceleration signals, deep learning, convolutional neural networks

## Abstract

Sensor- orientation is a critical aspect in a Human Activity Recognition (HAR) system based on tri-axial signals (such as accelerations); different sensors orientations introduce important errors in the activity recognition process. This paper proposes a new preprocessing module to reduce the negative impact of sensor-orientation variability in HAR. Firstly, this module estimates a consistent reference system; then, the tri-axial signals recorded from sensors with different orientations are transformed into this consistent reference system. This new preprocessing has been evaluated to mitigate the effect of different sensor orientations on the classification accuracy in several state-of-the-art HAR systems. The experiments were carried out using a subject-wise cross-validation methodology over six different datasets, including movements and postures. This new preprocessing module provided robust HAR performance even when sudden sensor orientation changes were included during data collection in the six different datasets. As an example, for the WISDM dataset, sensors with different orientations provoked a significant reduction in the classification accuracy of the state-of-the-art system (from 91.57 ± 0.23% to 89.19 ± 0.26%). This important reduction was recovered with the proposed algorithm, increasing the accuracy to 91.46 ± 0.30%, i.e., the same result obtained when all sensors had the same orientation.

## 1. Introduction

In the last decade, there has been an increasing interest in Human Activity Recognition (HAR) for recognizing the physical activities that people perform during their day-to-day lives. State-of-the-art systems for HAR integrate different signal processing and deep learning techniques [1,2,3,4,5,6,7,8], reaching promising results in different applications, e.g., sports monitoring [9,10,11] (such as fitness tracking, training adaptation, or personal incentivizing), rehabilitation [12], and respiratory diseases spread minimization [13].

In these applications, sensor orientation is a critical aspect when using tri-axial signals (such as accelerations); different sensor orientations can introduce important errors in the activity recognition process. In real scenarios (not supervised by experts), people place their sensors in different orientations, compromising the system performance. For example, fit bands and smartwatches can rotate along the wrist, presenting very different orientations. This fact affects the final recognition performance, requiring strategies to reduce the negative impact of these changes.

State-of-the-art HAR systems include robust features for dealing with different sensor orientations, but they do not incorporate specific algorithms for correcting the mismatches in sensor orientation. This work proposes a preprocessing module to correct these mismatches by extracting a consistent reference system and transforming the tri-axial acceleration signals from the sensor reference system to this consistent reference system. The proposed algorithm increases the robustness of the HAR system against sensor orientation changes, obtaining important improvements over several state-of-the-art systems and datasets. The main contributions of this paper are as follows:

- A new preprocessing algorithm for mitigating the effects of sensor orientation variability. Firstly, this algorithm generates a consistent reference system from the estimation of gravitational and forward movement directions. Secondly, the tri-axial acceleration recorded from the sensor is transformed from the sensor reference system to the consistent reference system. This proposal has demonstrated robust activity recognition even when sudden and abrupt sensor orientation changes happened during data recording.
- A study of the effect of the proposed algorithm depending on the type of activity, i.e., movements or postures.
- The evaluation of the proposal using six well-known HAR systems and datasets in a subject-wise cross-validation scenario, including a wide variety of subjects, activities, devices, and locations.

This paper is organized as follows. Section 2 reviews the literature, discussing different previous works related to the topic of this work. Section 3 describes the materials and methods used, including the system architecture, the proposed algorithm for adapting the sensor orientation through a consistent reference system, the signal processing and deep learning approaches, and the evaluation details. Section 4 describes the datasets and discusses the experiments and the obtained results. Finally, Section 5 summarizes the main conclusions of the paper.

## 2. Related Works

In the HAR field, the use of wearable devices is widely extended [14,15,16]. HAR systems based on tri-axial inertial signals (such as accelerations) have an important problem regarding the sensor orientations. In real scenarios (not supervised by experts), people place their sensors in different orientations (fit bands and smartwatches can rotate along the wrist), compromising the system performance.

In the literature, there are few proposals dealing with different sensor orientations [17]. Most of these studies have focused their contributions on extracting robust features (such as the magnitude of the accelerometer vector [18] or specific vertical and horizontal features [19]) that are not sensitive to orientation changes. These features showed good robustness, but they did not obtain the best performance. In the same way, San-Segundo et al. [20] used different mitigation techniques to deal with heterogeneities in HAR using smartphones and smartwatches. They used different feature extraction strategies such as filtering or session-specific normalization of feature data.

Only two previous works have been identified which propose similar algorithms to compute consistent axes for representing tri-axial acceleration. In the first study, Henpraserttae et al. [21] estimated the vertical and forward–backward axes of the body in several movements. These initial experiments were very promising, but the experimental setup only included recordings from five subjects and six daily activities, using data from the same subjects to train and test their system. In a real application, the system must be tested with different subjects compared to those used in training. The second work [22] developed two methods to remove the effect of absolute sensor orientation from the raw sensor data: a heuristic transformation and a singular value decomposition-based transformation. These techniques did not provide significant improvements for reducing the degradation due to wrong sensor orientation.

This paper solves the limitations of these previous works, proposing a new algorithm for estimating a full consistent reference system (composed of three main axes) and transforming tri-axial acceleration from the sensor system to the consistent reference system. This preprocessing algorithm has been evaluated on state-of-the-art HAR systems considering six different datasets in realistic scenarios (training and testing with different subjects). This work covers the research gap between these preliminary studies and the proposal of a full solution evaluated with several datasets in realistic scenarios.

In order to complete the related work section, it is interesting to comment that the analysis of acceleration signals has been applied to other fields such as driving [23]. In this work, deep learning techniques have been used to estimate the vehicle movement direction. This forward movement directional vector is useful for multiple applications such as characterizing driving styles or detecting dangerous events. In addition, some previous works [24,25,26] have performed IMU-based attitude estimation in automotive and human–robotic interaction systems. In fact, the authors have analyzed the effects of the bias and error of the IMUs and introduce other signals to improve the attitude estimation. Regarding driving applications, there also exist previous works [27] that have been focused on vehicle trajectory extraction, reconstruction, and evaluation to develop automated driving systems.

## 3. Materials and Methods

This section includes information about the proposed algorithm to adapt the acceleration coordinates to a consistent reference system, the architecture of the state-of-the-art system (describing the main modules), and the evaluation of the systems in different scenarios.

### 3.1. System Architecture

**Figure 1:**
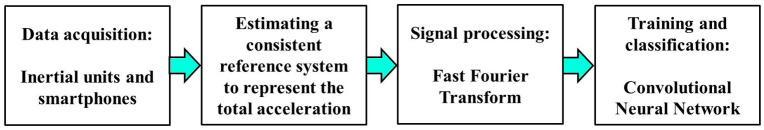
System architecture including the new algorithm before signal processing.

Figure 1 shows a general module diagram of an HAR system, incorporating the new algorithm as a previous step before the signal processing. Once the acceleration data are obtained from inertial units or smartphones, the consistent reference system is obtained. Then, the original acceleration is transformed from the sensor coordinates to this consistent reference system. This new representation is independent of the sensor orientation. Afterward, it is possible to apply signal processing techniques such as Fast Fourier Transform to extract relevant information from the motion signals in the frequency domain. Finally, these spectra are included in a Convolutional Neural Network to model and classify the different physical activities. This architecture has been used to obtain state-of-the-art performance in the datasets considered in this study.

### 3.2. Estimating a Consistent Reference System to Represent the Total Acceleration

**Figure 2:**
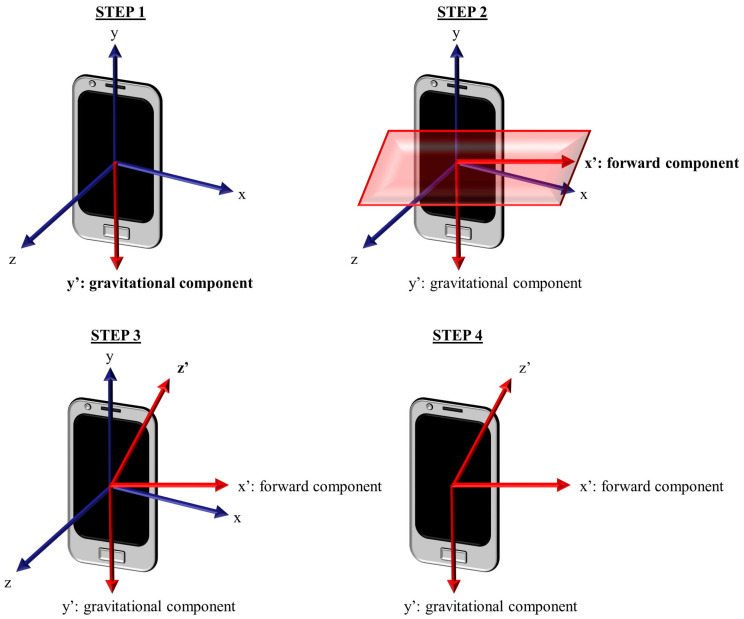
Generation of the consistent reference system through the algorithm. (Red part of Step 2 Maybe you could say that it represents the plane perpendicular to gravitational component that contains forward component.)

The proposed approach aims to create a consistent reference system independently of the sensor orientation and represent the total acceleration according to this consistent reference system. This process is composed of the following main steps (see Figure 2):

1. Firstly, the gravity vector is estimated from the total acceleration recorded from the accelerometer. Each coordinate of gravity is computed by applying a sliding mean over the three coordinates (X, Y, and Z in the sensor reference system) of the total acceleration through a convolution operation. Computing the average, we remove subject movements, leaving only the gravity [28]. For this step, a sliding mean filter of 5 s was used to compute the mean over each gravitational coordinate. In this way, we obtain the three components of gravity at each sample point. The filter size was analyzed in preliminary experiments, but it did not affect the results.
2. Secondly, we obtain the horizontal acceleration by subtracting the component in the gravity (vertical) direction from the total acceleration vector. After subtracting the vertical component, we compute the forward direction at each sample point by applying a sliding mean (5 s) over the horizontal acceleration. Unit vectors in the gravitational and the forward directions are computed dividing the original vectors by their magnitudes.
3. Thirdly, to complete the three axes system, the third unit vector is computed (at each sample point) as the cross product of gravitational and forward unit vectors.
4. Finally, the algorithm computes the new coordinates of the total acceleration according to this new reference system. The transformation of the total acceleration coordinates from the sensor reference system to the consistent reference system is accomplished by using Equation (1), where the sub-index “new” denotes the consistent reference system, and the sub-index “orig” refers to the sensor reference system. For example, $u_{x\_new}$ refers to the x coordinate of the new reference system, while $u_{x\_orig}$ refers to the *x* coordinate of the sensor reference system. In this equation, $V_{x\_\mathrm{new}}$, $V_{y\_\mathrm{new}}$, and $V_{z\_\mathrm{new}}$ are the acceleration coordinates according to the consistent reference system, and $V_{x\_\mathrm{orig}}$, $V_{y\_\mathrm{orig}}$ and, $V_{z\_\mathrm{orig}}$ are the acceleration coordinates respect to the sensor reference system. The unit vectors of the consistent reference system are the forward (*x*), gravitational (*y*), and cross-based computed direction (*z*) vectors. To transform the acceleration from one reference system to another, it is necessary to use the three coordinates of the unitary vector for both sensor and consistent reference systems. These coordinates are referred to the sensor reference system. These unitary vectors (and their coordinates) are used to compute the elements of the transformation matrix as shown in Equation (1).
  (1) $$\left[\begin{array}{c} V_{x\_new}\\ V_{y\_new}\\ V_{z\_new} \end{array}\right]=\left[\begin{array}{ccc} u_{x\_new}\cdot u_{x\_orig} & u_{x\_new}\cdot u_{y\_orig} & u_{x\_new}\cdot u_{z\_orig}\\ u_{y\_new}\cdot u_{x\_orig} & u_{y\_new}\cdot u_{y\_orig} & u_{y\_new}\cdot u_{z\_orig}\\ u_{z\_new}\cdot u_{x\_orig} & u_{z\_new}\cdot u_{y\_orig} & u_{z\_new}\cdot u_{z\_orig} \end{array}\right]\left[\begin{array}{c} V_{x\_orig}\\ V_{y\_orig}\\ V_{z\_orig} \end{array}\right]$$

Figure 2 shows the evolution of the algorithm, including the representation of the motion vector based on the sensor reference system and the generation of the consistent reference system.

This proposed approach computes the forward-direction vector of the acceleration signal to build the consistent reference system. The computation of the consistent reference system requires an additional computational overhead of 10%, but it does not affect the real time nature of the system. Even this additional computation overhead is reached when including the computation of the consistent reference, the real-time inference time was RT < 10^−2^ (1 h of signal is inference in of than 10 s).

This algorithm works for movements such as running or cycling, but it has an important limitation when dealing with posture classification. When a person is, for example, sitting or standing, there is no motion in forward direction (this vector is zero), so it is not possible to compute any consistent reference system. To solve this limitation, we have used the gravitational component as a reference: in the case of postures, instead of using the described algorithm, we directly subtracted the component of the gravity direction from the total acceleration signals of the postures. The gravity is computed from movements, not from postures, so it was necessary to have an initial module for separating postures and motion activities as we will see later.

### 3.3. Signal Processing and Deep Learning Approaches

After the new preprocessing, a signal processing module divides the recording signals into analysis windows and compute the spectrum of each window. The posterior classification module aims to identify the activity at each window. The system segments the physical activity recordings using overlapped Hanning windows of 5 s using a step of 1 s. This configuration has been successfully used in previous works [2,29,30] reporting state-of-the-art performance. 

After windowing, we compute the Fast Fourier Transform to generate the spectrum of each window in a range of frequencies between 0 and 10 or 20 Hz depending on the sampling frequency of the dataset. The magnitude bins of the spectrum are the input to the deep neural network. The use of FFT coefficients is justified because these features offered better results than time domain sequences or time domain features in previous works [20,31].

In state-of-the-art HAR systems, the recognition module is based on deep learning algorithms such as CNN [3,29]. The best deep learning architecture proposed in previous works [2,29], and also used in this work, is composed of two main parts: a feature learning subnet and a classification subnet. The first subnet learns features from window spectra, using two convolutional layers with intermediate max pooling layers. The second subnet uses fully connected layers to classify the learned features as a predicted activity. The architecture includes layers after max pooling and fully connected layers to avoid overfitting during training. The last layer uses a SoftMax activation function to offer the predictions of each activity for every analysis window, while intermediate layers use ReLU for reducing the impact of the gradient vanishing effect. We use categorical cross-entropy as the loss metric and the root-mean-square propagation method as the optimizer. This deep neural network has been used to model the data from all the analyzed datasets, using a different number of neurons in the last layer depending on the number of classes in each dataset.

**Figure 3:**
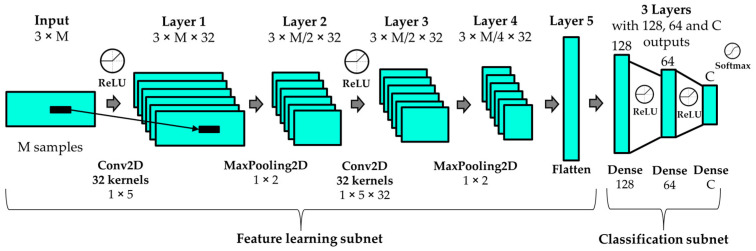
Convolutional Neural Network architecture used in this work for all the datasets.

Figure 3 represents the architecture used in this work to model and classify the physical activity of all the datasets, where M denotes the number of samples for each signal (corresponding to the magnitude bins of the spectra) and C indicates the number of recognized activities. 

### 3.4. Evaluation Setup

In this work, we have considered a subject-wise cross-validation, which is a version of the k-fold cross-validation procedure where the folds contain recordings from different subjects. In this methodology, the data are divided into k groups or folds to train, validate, and test the system with different data. However, it is assured that all the recordings from the same subject are included only in a fold. In this case, a subset of subjects is used for testing, and data from different subjects are used for training and validation in each iteration. The system is trained, validated, and tested with recordings from different subjects. Once the system model is fitted on the training subset, the validation subset is used for optimizing the model hyperparameters. Finally, the system is evaluated with the testing subset. This process is repeated several times, leaving different subjects for testing in each iteration. The results are the average of the results obtained for all trials (10 folds in this work). This methodology simulates a real-life scenario where the system is evaluated with recordings from subjects different from those used for training.

As evaluation metrics, we have used accuracy, which is defined as the ratio between the number of correctly classified samples and the number of total samples. Considering a classification problem with N testing samples and C classes, accuracy is defined in Equation (2), where $P_{\mathrm{ii}}$ refers to the elements of the principal diagonal in the confusion matrix.

(2) $$\mathrm{Accuracy}=\frac{1}{N}\overset{C}{\underset{i=1}{\sum }}P_{\mathrm{ii}}$$

Confidence intervals are used to show statistical significance values and provide confidence about the reliability of the results. These intervals include plausible values for a specific metric. We will assure that there exists a significant difference between the results of two experiments when their confidence intervals do not overlap. Equation (3) represents the computation of confidence intervals attached to a specific accuracy value and N samples when the confidence level is 95%.

(3) $$CI\left(95\%\right)=\pm 1.96\sqrt{\frac{accuracy\;\cdot \left(100-accuracy\;\right)}{N}}$$

## 4. Results and Discussion

This section describes the datasets used in this study, the experimental setups, the results, and the main discussions.

### 4.1. Datasets

For this work, we have used six publicly available HAR datasets. The combination of these datasets contains different sensing devices and a wide variety of physical activities, including repetitive movements such as running or walking and postures such as sitting and standing. The datasets used are WISDM_lab (Activity Prediction) [32], WISDM_wild (Actitracker) [33], MotionSense [34], USC-HAD [35,36], PAMAP2 [37], and HARTH [38].

The WISDM_lab dataset contains physical activity from 36 subjects that were carrying a smartphone in their front pants leg pocket. The recording device included an embedded accelerometer sampling at 20 Hz. The data collection was supervised by one of the laboratory team members to ensure the quality of the data. The subjects performed the following activities: walking, jogging, ascending stairs, descending stairs, sitting, and standing. As the samples from ascending and descending stairs were limited, both classes were joined as one: stairs activity. This dataset includes more than 15 h of recorded activity.

The WISDM_wild dataset includes physical activity recordings performed by 209 subjects while wearing a smartphone (HTC Evo model) with an accelerometer sensor using a sampling frequency of 20 Hz. The dataset contains in-the-wild data because the recordings were collected in real conditions without expert supervision. They labeled the data through a drop-down data label chooser in an application. In this context, there were no restrictions about where to wear the device, so the subjects could record the activity while keeping the smartphone inside a shirt pocket or a trousers pocket, even while holding it in the hand. The subjects performed the following activities: walking, jogging, stairs, sitting, standing, and lying down. This dataset includes 40 h of recorded activity.

The MotionSense dataset contains recordings of different physical activities performed by 24 subjects at the Queen Mary University of London’s Mile End campus. These participants wore in their trousers’ front pocket a smartphone (iPhone 6S model) with an accelerometer sampling at 50 Hz. The subjects performed the following activities: walking downstairs, walking upstairs, sitting, standing, walking, and jogging. This dataset contains 8 h of recordings.

The USC-HAD dataset includes recordings from 14 subjects performing physical activities while wearing an IMU (MotionNode, online: https://sipi.usc.edu/had/mi_ubicomp_sagaware12.pdf, available on 11 June 2023) packed into a pouch and attached to the front right hip. In this case, the sensor orientation variability could be lower thanks to the attachment. This measurement unit included an accelerometer sampling at 100 Hz. The physical activities included in this dataset are walking forward, walking left, walking right, walking upstairs, walking downstairs, running forward, jumping, sitting, standing, sleeping, elevator up, and elevator down. This dataset includes approximately 8 h of recorded activity.

The PAMAP2 dataset contains recordings of different physical activities performed by nine people wearing three IMUs (Inertial Measurement Units) (Trivisio, Yutz, France) with accelerometers sampling at 100 Hz. These units are placed onto three different body locations: chest, wrist on the dominant arm, and ankle on the dominant side. In this case, the sensor orientation variability could be lower thanks to the attachments. The subjects performed the following activities: lying, sitting, standing, walking, running, cycling, Nordic walk, ascending stairs, descending stairs, ironing, vacuum cleaning, and rope jumping. This dataset includes more than 5.5 h of recorded activity.

The HARTH dataset includes recordings of different physical activities performed by 22 people wearing two accelerometer sensors (Axivity AX3 model) sampling at 100 Hz. These units are placed onto the right front thigh (approximately 10 cm above the upper kneecap) and lower back (approximately third lumbar vertebra). This dataset contains data collected under laboratory conditions and in a free-living setting where no further instructions on where and when to carry out the activities but including the sensor attachments. In this case, the sensor orientation variability could be lower thanks to the attachments. The physical activities included in this dataset are walking, running, shuffling (standing with leg movement), ascending stairs, descending stairs, standing, sitting, lying, cycling while sitting, and cycling while standing. The recordings of inactive cycling while sitting (without leg movement) and inactive cycling while standing (without leg movement) were not used in this work because these activities are too unusual. This dataset includes more than 17 h.

Table 1 includes the main characteristics of the datasets used in this work, including the number of subjects, the number of physical activities (repetitive movements and postures), the devices used for recording the motion, their location, and the sampling frequency of the accelerometers.

Table 2 displays the overall duration of recorded physical activity for the datasets used in this work, as well as the time for each specific physical activity within each dataset.

### 4.2. Experimental Setups and Results

Some of our HAR previous works were focused on optimizing the different modules of the HAR system obtaining the highest recognition performance over PAMAP2 [2,3], MotionSense [39], or USC-HAD [1] datasets. This work is focused on showing how the proposed algorithm could correct the recognition errors due to changes in the recording sensor orientation. The hypothesis to demonstrate in these experiments is that the proposed algorithm can compensate the degradations suffered by state-of-the-art HAR systems when random rotations are introduced in the sensors. The goal is to recover the system degradation, obtaining the same performance compared to the baseline experiment (where all the sensors were properly oriented). We considered four different experimental setups or situations to demonstrate this hypothesis:

1. Baseline. First, we used the original data from the datasets for training and testing a state-of-the-art HAR system. Most of the datasets (except for WISDM_wild) were obtained under laboratory conditions; the data collection protocol was controlled by experts and all the recording devices were located using the same orientation; thus, there was no effect due to sensor orientation.
2. Rotated. Second, we included random rotations over the tri-axial accelerometer signals to simulate changes in sensor orientation. These changes were based on the rotation matrix, which performed a transformation in Euclidean space. Since we managed tri-axial signals, we applied the rotation over one out of the three axes that were randomly selected for each subject, keeping the remaining axes fixed. The rotation matrices used for each axis are included in Equation (4). We performed preliminary studies using different angle values, but no effect was observed, so we finally applied a rotation of θ equal to 45° for this work.
  (4) $$R_{x}\left(\theta \right)=\left[\begin{array}{ccc} 1 & 0 & 0\\ 0 & \cos\left(\theta \right) & -\sin\left(\theta \right)\\ 0 & \sin\left(\theta \right) & \cos\left(\theta \right) \end{array}\right];\;R_{y}\left(\theta \right)=\left[\begin{array}{ccc} \cos\left(\theta \right) & 0 & \sin\left(\theta \right)\\ 0 & 1 & 0\\ -\sin\left(\theta \right) & 0 & \cos\left(\theta \right) \end{array}\right];\;R_{z}\left(\theta \right)=\left[\begin{array}{ccc} \cos\left(\theta \right) & -\sin\left(\theta \right) & 0\\ \sin\left(\theta \right) & \cos\left(\theta \right) & 0\\ 0 & 0 & 1 \end{array}\right]$$
3. Rotated and algorithm. Third, we applied the proposed algorithm to compensate for the random sensor rotations by extracting a consistent reference system and transforming the acceleration from the sensor reference system to the consistent reference system. The same algorithm was applied to all types of activities, including movements and postures.
4. Rotated and algorithm per type of activity. Finally, we repeated the third experimental setup but applying specific approaches depending on the type of activity (movements or postures). We used the approach based on the consistent reference system to movements and the solution of subtracting the gravity for postures.

**Figure 4:**
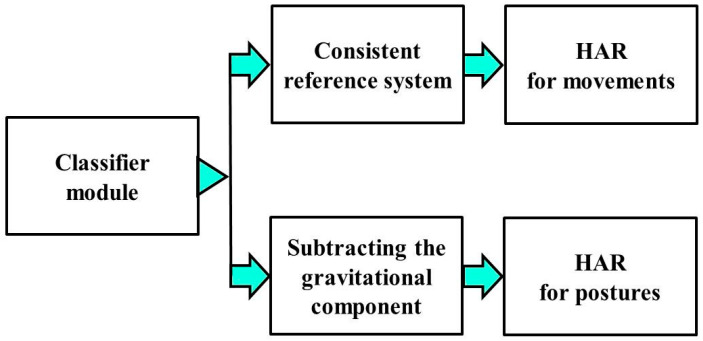
HAR system dealing with different types of activities.

In cases of developing an HAR system dealing with several types of activities, it would be necessary to include an initial classifier module [2] to distinguish between movements and postures and then apply specific approaches based on the type of activities (computing the new reference system or just subtracting the component of the gravity direction), as shown in Figure 4. Considering this system, an initial classifier was included to automatically detect the type of activity: movements vs. postures. This module was implemented using the same CNN architecture presented in Section 3.3, but with a SoftMax function at the end to classify two classes: movements and postures. This classifier has a very high average accuracy (over 95%) because it deals with a simple classification task. Afterward, each example is processed in a different way depending on this classification. The results presented in this work also include the errors produced by this automatic pre-classification.

In the case of processing each type of activity separately, we applied the most advantageous approach for each case as mentioned in the algorithm description section. For movements (they have a forward movement direction), we generated a consistent reference system based on the person’s movement direction. For postures (as they do not have a motion forward direction), we directly subtracted the gravitational component. 

Table 3 includes the accuracy of the experiments in the four experimental setups for all the datasets. Table 3 already includes the impact of the initial classification module to distinguish between movements and postures. We observe that the performance of the results when considering miss-oriented sensors (column “Rotated” of Table 3) decreased significantly compared to the baseline system (column “Baseline” of Table 3); including random rotations over the acceleration signals makes the system performance decrease. For example, in the case of WISDM_lab dataset, we initially reached an accuracy of 91.57 ± 0.23% and decreased to 89.19 ± 0.26% when including random rotations.

In a first attempt, we used the same algorithm for all the activities (column “Rotated and algorithm” of Table 3). We were able to recover part of the degradation, but it was not possible to reach the same accuracy obtained in the baseline (without random rotations). For WISDM_lab dataset, we obtained an accuracy of 90.28 ± 0.25%. Extracting a consistent reference system with postures can be counterproductive because it cannot be extracted properly.

In a second attempt, we decided to apply specific approaches depending on the type of activity: the consistent new reference system for repetitive movements and subtracting the component of the gravity direction for postures. In this case (column “Rotated and different approaches per type of activity” of Table 3), we were able to reach a similar performance to the baseline experimental setup. For example, in the case of WISDM_lab dataset, we obtained an accuracy of 91.83 ± 0.23% that was reduced to 91.36 ± 0.24% when including the initial classifier module (with a 99.90 ± 0.03% of classification accuracy). Adapting the algorithm to the type of activity solved the degradation from sensor orientation. In the case of WISDM_wild, we not only recovered the degradation but also achieved an important improvement in performance compared to the baseline system. One of the reasons for this improvement is that this dataset already contains real data whose collection protocol was not supervised by experts, so the original data were already affected by some rotations that were mitigated by our proposed approach. Only a slight reduction of performance (2.37%) was obtained by comparing the baseline and rotated setup, which means that the original data were noisy and already included sensor rotations.

Table 4 includes the results of the experiments for all the datasets distinguishing types of activity. Results suggest that the errors due to the changes in sensor orientation could be mitigated by applying specific algorithms because the final systems could attain a similar performance compared to the baseline.

**Figure 5:**
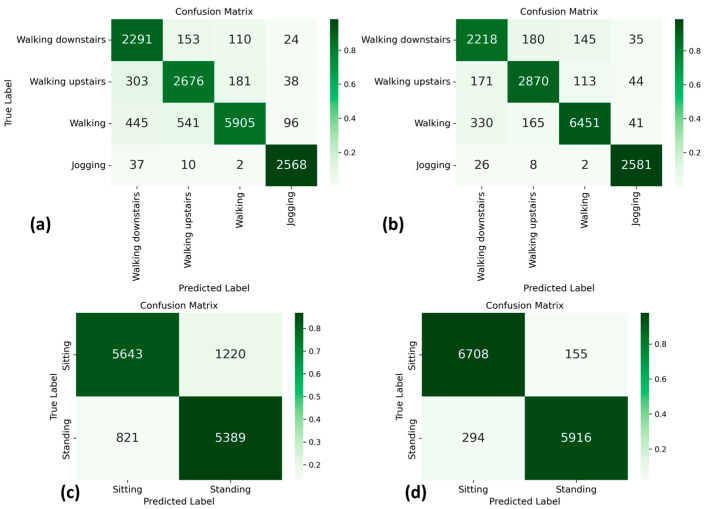
Confusion matrices for repetitive movements and postures classification results in the MotionSense dataset, using rotated experimental setup (**a**,**c**) and rotation and algorithm per type of activity experimental setup (**b**,**d**).

Figure 5 displays four confusion matrices for the MotionSense dataset by which to compare different experimental setups. These matrices show the classification results in the rotated setup for the repetitive movements (Figure 5a, related to 87.39 ± 0.52% of accuracy) and postures (Figure 5c, related to 87.39 ± 0.52% of accuracy). Additionally, we show confusion matrices for the rotation and algorithm per type of activity experimental setup: repetitive movements (Figure 5b, related to 91.81 ± 0.43% of accuracy) and postures (Figure 5d, related to 96.57 ± 0.31% of accuracy). It is possible to observe that the confusion for the different classes is reduced when applying our algorithm. 

Table 5 details the precision and recall results per activity for these experiments, where it is observed that both metrics of every class, including repetitive movements and postures, increase when applying the algorithm, except for recall of walking downstairs. However, it is important to mention that this class is compared to very similar activities, such as walking upstairs, walking, and jogging.

### 4.3. Discussions and Insights

The first insight obtained from results in Table 3 was the important degradation suffered by state-of-the-art HAR systems when random sensor rotations are introduced in the tri-axial accelerations. This important degradation justified the main contribution of this study, namely, the proposal and evaluation of a new pre-processing algorithm to compensate for sensor rotations in state-of-the-art HAR systems. 

The proposed algorithm builds a consistent reference system independently of the sensor orientation and represents the acceleration according to this consistent reference system (independently of the sensor orientation). The creation of the consistent reference system is based on the estimation of gravitational and forward movement directions. These orthogonal directions (plus the cross product) form a tri-axial orthogonal system. The acceleration representation is transformed from the sensor axial system to this new reference system, which is more consistent in its movements.

During the experiments, we realized that this algorithm works well for movements such as running or cycling but not for postures such as sitting or standing. In postures, there is no motion in the forward direction, so it is not possible to extract the new reference system. As an alternative, we proposed subtracting the gravity from the total acceleration. This insight was very important because it allowed the combination of several strategies and the design of a complete solution (applicable to all types of activities).

This complete solution has been evaluated with state-of-the-art HAR systems based on deep learning algorithms over six different datasets. These datasets cover a very wide range of activities, subjects, and recording conditions. The experiments section showed the results of four different situations: the baseline situation (where all the sensors were correctly oriented), the rotated scenario (with the introduction of random rotations to the sensors), and two applications of the proposed algorithm (i.e., same strategy for all the activities or differentiating between movements and postures). From the experiments in Table 3, we can conclude that the proposed method is able to recover the degradation produced in the HAR systems when random rotations are introduced in the tri-axial acceleration. This recovery is complete when we apply a different strategy depending on the type of activity (movements or postures). This result is very important because this is the first work proposing a complete solution (for any kind of activity). Another insight obtained from the results is the less degradation in movements compared to postures when including sensor rotations (Table 4): postures are more sensible to changes in sensor orientation.

To compare our system with previous works, we computed the mitigation capability as the percentage of degradation which the algorithm can recover or compensate. The previous work with the best mitigation capability was [19], extracting vertical and horizontal features. The best proposed method was able to recover 86% of the performance decrease due to sensor rotation. In our case, the algorithm proposed in this paper recovered (on average, along six different datasets) 100% of the performance decrement. We observed that the algorithm proposed in this paper was able to deal with severe sensor rotations, especially in locations such as the wrist for the PAMAP2 dataset (see the improvement of 6.19% comparing rotated and last columns in Table 3). 

## 5. Conclusions

Changes in sensor orientation are an important problem that affects the performance of HAR systems in many different applications. In this paper, a new preprocessing algorithm has been proposed to reduce the negative impact of these changes. This algorithm creates a consistent reference system (based on the estimation of gravitational and forward movement directions) and transforms the tri-axial accelerometer signals representation. This algorithm has been very useful for movements; in this case, it is easy to leverage the gravitational and forward component information to create a consistent reference system with which to represent the movement. In the case of postures (sitting or standing), a forward movement vector does not exist, and it cannot be used for extracting the consistent reference system. In these cases, subtracting the gravitational component of the signals has been more useful.

The proposed approach was included in a preprocessing module (i.e., before the signal processing module) of a state-of-the-art HAR system and evaluated over six different HAR datasets that include repetitive movements and postures. We used a subject-wise cross-validation methodology: different subjects were used for training, validation, and testing the system in each iteration. For the WISDM dataset, the sensor orientation errors reduced the classification accuracy from 91.56 ± 0.23% to 89.19 ± 0.26%. This performance decrease was mitigated with the proposed algorithm, increasing the accuracy to 91.46 ± 0.30% when applying specific approaches depending on the type of activity, and reaching the same results that those achieved with the sensor correctly oriented.

However, this study has a limitation: the current algorithm is applied over isolated sensors, so it could be interesting to deal with several sensors at the same time. An interesting solution could be to estimate the consistent reference system from one sensor and then use this system for all the sensors. Another interesting future work could be the analysis of the best sensor location to estimate the consistent reference system. Finally, we would like to apply the proposed algorithm to data with real device orientation changes.

## Figures and Tables

**Table 1:** Main characteristics of the datasets used.

Dataset	# Subject	# Activity	# Rep. Mov.	# Posture	Device	Device/Sensor Location	Sampling Rate (Hz)
WISDM_lab	36	5	3	2	Smartphone	Front pants pocket	20
WISDM_wild	209	6	3	3	Smartphone	Free	20
MotionSense	24	6	4	2	Smartphone	Front pants pocket	50
USC-HAD	14	12	7	5	Sensor	Front right hip	100
PAMAP2	9	12	9	3	Sensor	Hand, chest, and ankle	100
HARTH	22	10	7	3	Sensor	Thigh and lower back	100

**Table 2:** Time for each activity within the datasets used characteristics of the datasets used.

Dataset	Total Time (h)	Time per Activity
WISDM_lab	15	Walking (20,970 s), jogging (16,453 s), stairs (11,063 s), sitting (2954 s), and standing (2306 s)
WISDM_wild	40	Walking (60,684 s), jogging (21,813 s), stairs (2515 s), sitting (32,607 s), standing (14,030), and lying down (13,424 s)
MotionSense	8	Walking downstairs (2578 s), walking upstairs (3198 s), sitting (6863 s), standing (6210 s), walking (6987 s), and jogging (2617 s)
USC-HAD	8	Walking forward (3772 s), walking left (2588 s), walking right (2755 s), walking upstairs (2118 s), walking downstairs (1974 s), running forward (1765 s), jumping (1072 s), sitting (2615 s), standing (2360 s), sleeping (3750 s), elevator up (1653 s), and elevator down (1602 s)
PAMAP2	5.5	Lying (1925 s), sitting (1852 s), standing (1899 s), walking (2387 s), running (978 s), cycling (1646 s), Nordic walk (1881 s), ascending stairs (1173 s), descending stairs (1051 s), ironing (1755 s), vacuum cleaning (2387 s), and rope jumping (488 s)
HARTH	17	Walking (11,661 s), running (2917 s), shuffling (standing with leg movement) (1180 s), ascending stairs (817 s), descending stairs (740 s), standing (7327 s), sitting (29,003 s), lying (4285 s), cycling while sitting (3965 s), and cycling while standing (544 s)

**Table 3:** Results of the four experimental setups for all the datasets. References are included with the descriptions of state-of-the-art HAR systems in each case.

Datasets and State-of-the-Art Systems	Accuracy (%) Depending on the Experimental Setup			
	Baseline	Rotated (Changes in Sensor Orientation)	Rotated and Algorithm	Rotated and Different Approaches per Type of Activity
WISDM_lab [32]	91.57 ± 0.23	89.19 ± 0.26	90.28 ± 0.25	91.36 ± 0.24
WISDM_wild [33]	73.54 ± 0.23	71.17 ± 0.23	71.52 ± 0.23	81.54 ± 0.20
MotionSense [39]	95.48 ± 0.24	88.22 ± 0.37	87.78 ± 0.38	92.09 ± 0.31
USC-HAD [1]	63.56 ± 0.56	58.62 ± 0.58	56.48 ± 0.58	59.35 ± 0.58
PAMAP2—Chest [2,3]	72.29 ± 0.63	65.35 ± 0.67	67.44 ± 0.66	68.12 ± 0.66
PAMAP2—Wrist [2,3]	77.27 ± 0.59	68.52 ± 0.65	70.06 ± 0.64	74.71 ± 0.61
PAMAP2—Ankle [2,3]	70.17 ± 0.64	63.46 ± 0.68	62.50 ± 0.68	67.93 ± 0.66
HARTH—Back [38]	88.58 ± 0.25	81.89 ± 0.30	80.21 ± 0.31	87.52 ± 0.26
HARTH—Thigh [38]	91.67 ± 0.22	83.90 ± 0.29	81.72 ± 0.30	87.56 ± 0.26

**Table 4:** Results of three experimental setups for the different datasets for each type of activity.

Dataset	Type of Activity	Accuracy (%) Depending on the Experimental Setup		
		Baseline (Supervised by Experts)	Rotated	Rotated and Algorithm per Type of Activity
WISDM_lab	Rep. Mov.	91.41 ± 0.25	88.64 ± 0.28	91.82 ± 0.24
	Postures	96.71 ± 0.48	71.67 ± 1.22	88.17 ± 0.87
WISDM_wild	Rep. Mov.	88.46 ± 0.21	89.18 ± 0.21	91.09 ± 0.19
	Postures	63.76 ± 0.38	47.46 ± 0.40	58.66 ± 0.39
MotionSense	Rep. Mov.	90.98 ± 0.45	87.39 ± 0.52	91.81 ± 0.43
	Postures	98.55 ± 0.21	84.39 ± 0.62	96.57 ± 0.31
USC-HAD	Rep. Mov.	59.72 ± 0.76	55.29 ± 0.77	58.66 ± 0.76
	Postures	67.75 ± 0.84	62.77 ± 0.87	67.19 ± 0.84
PAMAP2—Chest	Rep. Mov.	76.72 ± 0.71	63.38 ± 0.81	73.80 ± 0.74
	Postures	73.93 ± 1.14	57.91 ± 1.28	75.16 ± 1.12
PAMAP2—Wrist	Rep. Mov.	84.42 ± 0.61	73.23 ± 0.74	80.23 ± 0.67
	Postures	71.28 ± 1.18	57.33 ± 1.29	72.27 ± 1.16
PAMAP2—Ankle	Rep. Mov.	80.17 ± 0.67	75.97 ± 0.71	76.07 ± 0.71
	Postures	74.88 ± 1.13	54.88 ± 1.29	64.02 ± 1.25
HARTH—Back	Rep. Mov.	91.71 ± 0.37	88.20 ± 0.43	89.89 ± 0.40
	Postures	88.16 ± 0.31	84.62 ± 0.35	87.85 ± 0.32
HARTH—Thigh	Rep. Mov.	92.79 ± 0.34	87.46 ± 0.44	90.04 ± 0.40
	Postures	93.83 ± 0.23	84.29 ± 0.35	87.48 ± 0.32

**Table 5:** Precision and recall results per activity on two experimental setups for the MotionSense dataset.

Type of Activity	Activity	Results (%) Depending on the Experimental Setup			
		Rotated		Rotated and Algorithm per Type of Activity	
		Precision	Recall	Precision	Recall
Repetitive Movements	Walking downstairs	74.48 ± 1.54	88.87 ± 1.21	80.80 ± 1.47	86.04 ± 1.34
	Walking upstairs	79.17 ± 1.37	83.68 ± 1.28	89.05 ± 1.08	89.74 ± 1.05
	Walking	95.27 ± 0.53	84.51 ± 0.85	96.13 ± 0.46	92.33 ± 0.62
	Jogging	94.20 ± 0.88	98.13 ± 0.52	95.56 ± 0.78	98.62 ± 0.45
Postures	Sitting	87.30 ± 0.81	82.22 ± 0.90	95.80 ± 0.47	97.74 ± 0.35
	Standing	81.54 ± 0.94	86.78 ± 0.84	97.45 ± 0.40	95.27 ± 0.53

## Data Availability

The data presented in this study are openly available in different repositories: WISDM_lab (Activity Prediction) (https://www.cis.fordham.edu/wisdm/dataset.php), WISDM_wild (Actitracker) (https://www.cis.fordham.edu/wisdm/dataset.php), MotionSense (https://www.kaggle.com/datasets/malekzadeh/motionsense-dataset), USC-HAD (https://sipi.usc.edu/had/) [36], PAMAP2 (10.24432/C5NW2H), and HARTH (10.24432/C5NC90).
